# Supplementary material for: Distinct SARS-CoV-2 populational immune backgrounds tolerate divergent RBD evolutionary preferences
Source: Natl Sci Rev. 2024 Jun 5;11(7):nwae196. doi: 10.1093/nsr/nwae196 (PMC11275455; doi:10.1093/nsr/nwae196)
Supplement: nwae196_Supplemental_Files [file nwae196_supplemental_files.zip › Supplementary figures.pdf]

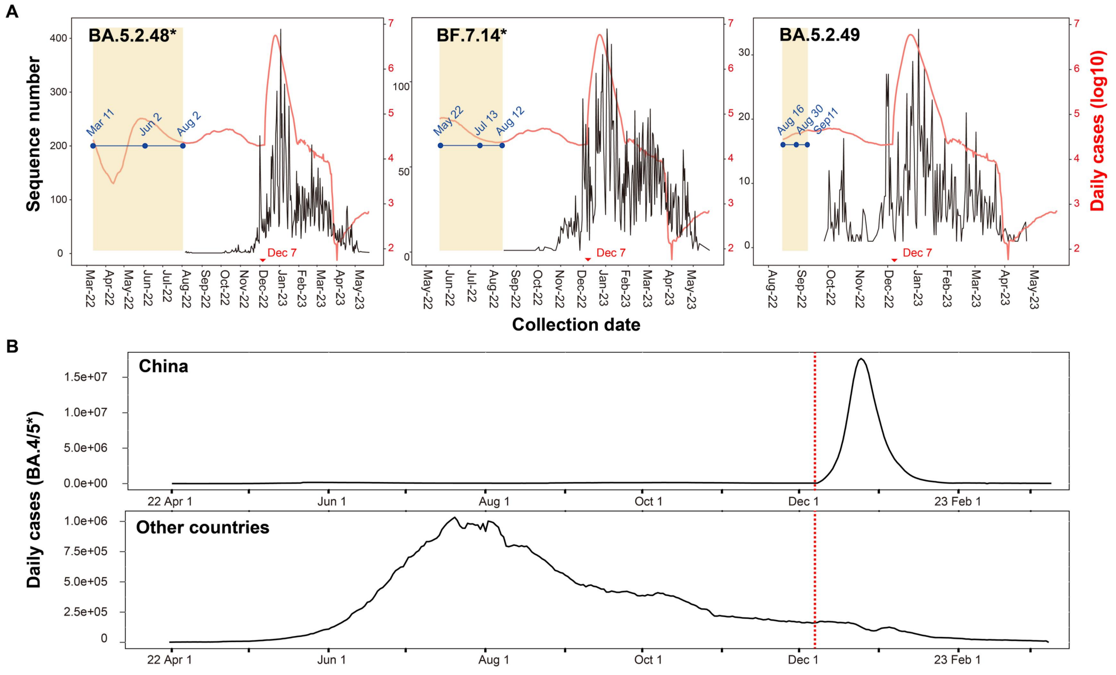


**Supplementary Fig. 1. The daily sequence numbers and cases.** A) The daily sequence numbers and cases of three Chinese lineages. The red line indicates the daily number of cases in China (right y-axis). The black line indicates the daily number of sequences collected in China that are uploaded to public databases (left y-axis). The occurrence time of the most recent common ancestor for each clade is inferred by BEAST and marked in blue, displaying both the median and the 95% confidence interval. The Pearson correlation coefficient between the number of daily sequence and the number of daily reported cases were 0.48, 0.44, and 0.41 ($p<0.0001$) for BA.5.2.48*, BF.7.14*, and BA.5.2.49. B) The daily cases of BA.4/5* in China and other countries. The number of cases was estimated from the total number of cases per day and the proportion of BA.4/5* estimated from the sequence data, and then averaged over 7 days. The red line indicates December 7.

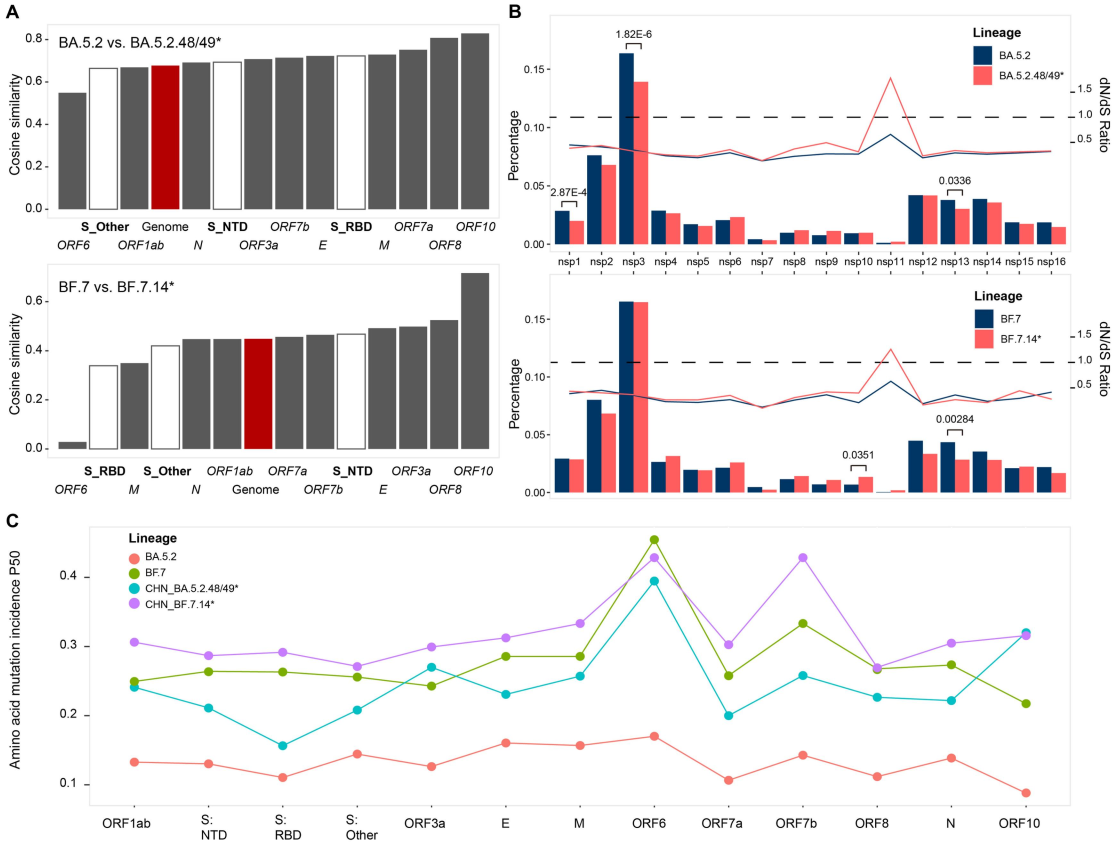


**Supplementary Fig. 2. Different in mutation incidence and distribution between the BA.5.2.48/49* and BF.7.14* lineages and their global counterparts.** A) Correlation of mutation incidence between the BA.5.2.48/49* and BF.7.14* lineages and their global counterparts. The cosine similarity was calculated based on the incidence of non-synonymous mutations in different genes of SARS-CoV-2. *S* gene was categorized into S_RBD, S_NTD, and S_Other in the analysis. B) Distribution of non-synonymous mutation events across sixteen nonstructural proteins (NSP) regions of the *ORF1ab* gene. The bar indicts the proportion of non-synonymous mutations (left y-axis) while the dots indicates the dN/dS ratio for each gene (right y-axis). The Bonferroni adjusted p-value was calculated by Fisher's exact test, with only statistically significant p-values (<0.05) are labeled in the figure. The sub-lineages

were not included in either the BA.5.2 or the BF.7 lineage. C) The amino acid mutation concentration across different genes. The P50 is the percentage of top prevalent mutations that account for half of the total mutation events. The S gene is divided into S:NTD, S:RBD, and S:Other regions.

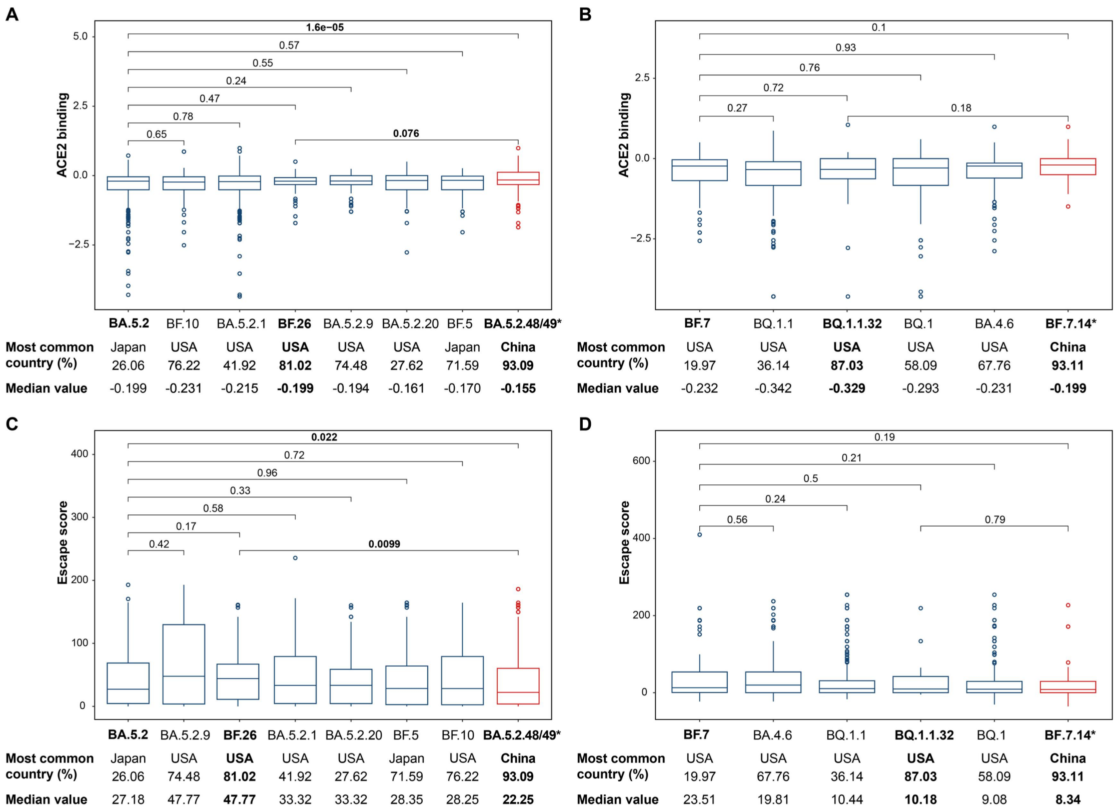


**Supplementary Fig. 3. The comparison of mutation escape scores and ACE2 binding scores between BF.7, BA.5.2, and other lineages.** A) Comparison of the ACE2 binding scores between the BA.5.2 and other lineages that have the same RBD sequence. B) Comparison of the ACE2 binding scores between BF.7 and other lineages that have additional mutations in the RBD compared to BA.5.2. C) Comparison of the escape score between BA.5.2 and other lineages that have the same RBD sequence. D) Comparison of the escape scores between BF.7 and other lineages that have additional mutations in the RBD compared to BA.5.2. Lineages were sorted by the median value. For each lineage, the median value of the score, the country that contributes the most sequences, and the proportion of sequences collected from the country were labelled at the bottom. Lineages that are primarily collected from a single country (>80% sequences) are labelled in bold.

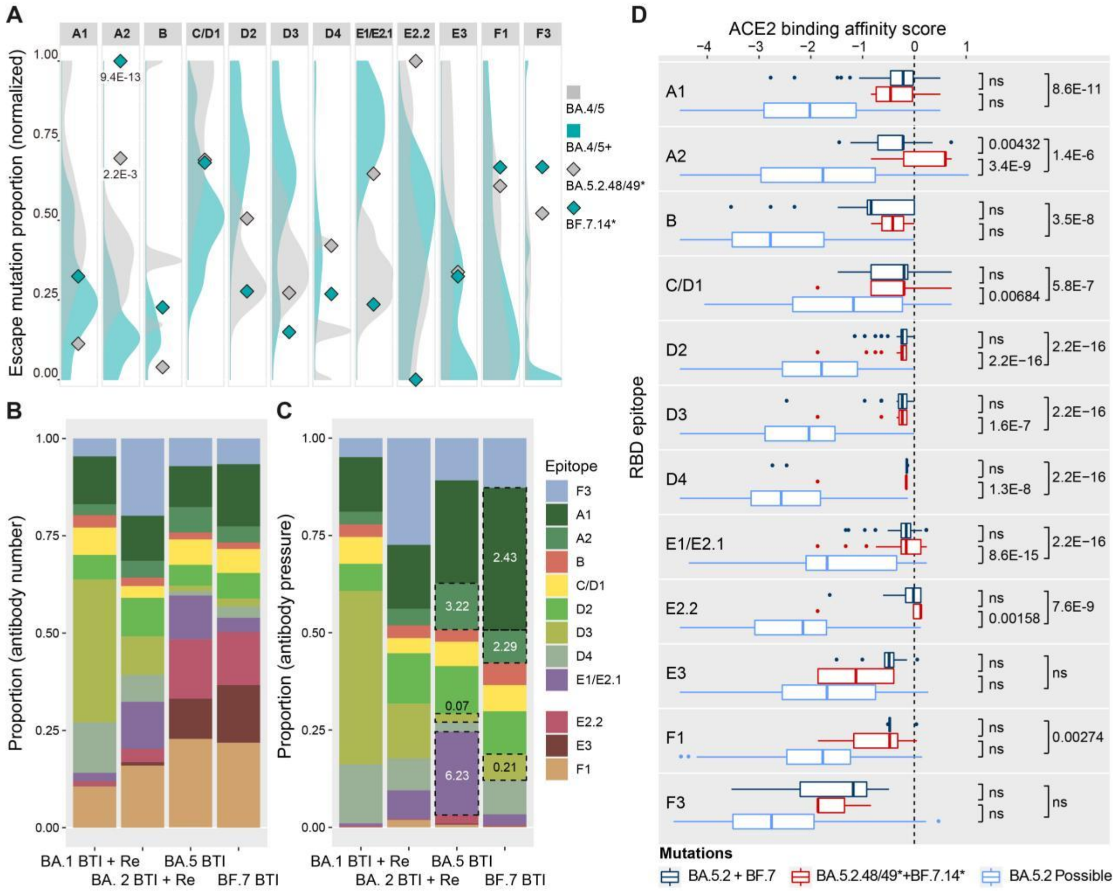


**Supplementary Fig. 4. The comparison of the property of escape mutations between the BA.5.2.48/49* and BF.7.14* lineages and global counterparts.** A) The proportion of escape mutations in different lineages. The proportion of escape mutations was normalized using the maximum value within each epitope. The density distribution was estimated using data from the BA.4/5 and BA.4/5+ groups, with the exclusion of the BA.5.2.48/49 and BF.7.14 lineages. The significance of the deviation in the escape score of BA.5.2.48/49 and BF.7.14 from the background distribution (assuming a normal distribution) was calculated as the probability of obtaining a value equal to or greater than the observed value, with only statistically significant p-values (<0.05) are labeled in the figure. B) The composition of the antibodies targeting different epitopes in convalescent sera with different infection histories. C) The

distribution of humoral immune pressure on different epitopes. Dotted boxes highlight epitopes with immune pressure alterations of over two-fold between convalescent sera from reinfection and breakthrough infection cases (the value within the box denotes the ratio of immune pressure in breakthrough infection sera to that in reinfection sera). Immune pressure on a specific epitope was calculated by summing the normalized neutralization IC50 values of the antibody that target the epitope. BA.1 BTI+Re: BA.1 breakthrough infection followed by reinfection with BA.5/BF.7; BA.2 BTI+re: BA.2 breakthrough infection followed by reinfection with BA.5/BF.7; BA.5 BTI: BA.5 breakthrough infection, BF.7 BTI: BF7 breakthrough infection. D) The ACE2 binding affinity score of escape mutations located in 12 RBD epitopes. Possible RBD mutations encompassed those caused by single-step nucleotide changes on the BA.5.2 genome (EPI_ISL_16614729). The center line indicates the median, the box represents the interquartile range (IQR), the whiskers extend to the furthest data point in each wing that is within 1.5 times the IQR, and the dots represents outliers. Bonferroni adjusted p-values were calculated using the Wilcoxon rank sum test. ns: not significant.
